# Collapsing Focal Segmental Glomerulosclerosis in a Patient with Acute Malaria

**DOI:** 10.1155/2015/420459

**Published:** 2015-07-05

**Authors:** Najamus Sehar, Emad Gobran, Suzanne Elsayegh

**Affiliations:** Staten Island University Hospital, 475 Seaview Avenue, Staten Island, NY 10305, USA

## Abstract

*Introduction*. Collapsing focal segmental glomerulosclerosis (FSGS) is most commonly seen in association with HIV infection. Rare data is available about the association between collapsing FSGS and malaria. *Case Description*. A 72-year-old African male patient presented to the hospital for generalized body aches, fatigue, fever, and night sweats for three days. He had history of recent travel to Ghana. Patient looked in acute distress and was shivering. Laboratory tests showed elevated serum creatinine (Cr) of 2.09 mg/dL (baseline was 1.5 mg/dL in 2012). Hospital course was significant for rapid elevation of Cr to 9.5 mg/dL and proteinuria of 7.9 grams. Autoimmune studies resulted negative. Blood smear resulted positive for *Plasmodium falciparum* and patient was treated with Artemether/Lumefantrine. Patient's fever and pain improved, but kidney function continued to deteriorate and he became oliguric. On day seven, he was started on Hemodialysis. Tests for different causes of glomerular pathology were also negative. He underwent left kidney biopsy which resulted in findings consistent with severe collapsing glomerulopathy. *Discussion*. This case illustrates a biopsy proven collapsing FSGS likely secondary to malarial infection requiring renal replacement therapy. Literature review revealed only few case reports that suggested the possible association of malaria with secondary form of FSGS.

## 1. Introduction

Collapsing glomerulopathy is a morphologic variant of FSGS characterized by segmental and global collapse of the glomerular capillaries, marked hypertrophy and hyperplasia of podocytes, and severe tubulointerstitial disease [1]. It is most commonly seen in association with HIV, but other numerous etiologies have been reported as well. These include infections like tuberculosis, cytomegalovirus, hepatitis B, and hepatitis C. Other causes include drugs such as bisphosphonates, steroids, opioids, and interferons; autoimmune diseases like SLE, adult Still's disease, and mixed connective tissue disease; and hematologic abnormalities including multiple myeloma, hemophagocytic syndrome, and acute monoblastic leukemia [1–5]. There have been very few cases of malaria associated collapsing glomerulopathy. Most of such cases are reported in Africa and Southeast Asia where malaria is more prevalent or amongst the immigrants from these areas. Malaria caused by* Plasmodium malariae* and* Plasmodium falciparum* is more commonly involved in acute renal failure as compared to other strains. Very few studies have been published, showing association of* P. falciparum* with collapsing FSGS.

## 2. Case Description

A 72-year-old man was admitted to hospital for right upper quadrant abdominal pain, generalized body aches, night sweats, chills, subjective fever at night, and weakness for three days.

One month before presenting, patient travelled to his native African country Ghana for a two-week trip. Ten days after returning back to the United States, he developed the symptoms. Initially, he took over the counter common cold medication without any relief. He denied sick contacts, vomiting diarrhea, shortness of breath, chest pain, or any other symptoms.

Patient was also taking mefloquine for malaria prophylaxis during his visit.

Patient's past medical history included hypertension treated for five years, high cholesterol, gout, and chronic kidney disease secondary to hypertension. The patient had no surgeries in past and had no known allergies. He denied smoking, alcohol intake, or drug abuse. Patient's home medications included allopurinol, losartan, amlodipine, labetalol, furosemide, and simvastatin.

On physical examination, temperature was 39.3 degrees of Celsius, blood pressure was 184/86 mm of Hg, respiratory rate was 18 breaths per minute, and heart rate was 76 beats per minute. Patient looked in acute distress and was shivering. He was awake, alert, and oriented to time, place, and person. Neck was supple. Lungs were clear and heart sounds regular on auscultation. No murmur, rubs, or gallop was appreciated. Abdomen was soft and nontender and bowel sounds were audible in all four quadrants. No leg edema was noted.

Initial blood tests in the emergency room showed a normal complete blood picture including white count with differential, platelet count and a hemoglobin of 12.3 grams/dL. On metabolic profile, patient's creatinine was found to be 2.09 mg/dL that was higher than baseline of 1.5 mg/dL recorded six months earlier. Urea nitrogen was 30 mg/dL with glomerular filtration rate of 38. Liver function tests were normal except for albumin of 2.6 g/dL and total bilirubin of 1.6 mg/dL. Creatinine kinase was 514 IU/L. Amylase was 54 U/L and lipase 35 U/L.

Initially, patient was admitted to medical floor and started on broad spectrum empiric antibiotics. Blood culture, urine cultures, and nasal swab for influenza were sent. Because of the acute kidney injury, furosemide and losartan were stopped and intravenous fluids were given. A urinary catheter was placed and urinalysis along with urine electrolytes was ordered.

On the second day of hospitalization, patient continued to have abdominal pain and fever. An abdominal computerized tomography scan without contrast showed findings concerning acute pancreatitis or acalculous cholecystitis.

On the third day, his serum creatinine rapidly increased to 4.17 mg/dL with GFR of 17. Urine analysis showed dark colored, cloudy, and concentrated urine with small amount of bilirubin, large blood, large protein, no white blood cells, and red blood cells of 3–5/hpf. No casts were seen on urine sediment. Urine electrolytes showed a fractional excretion of sodium of 1.2% and urine creatinine of 157 mg/dL. A urine protein to creatinine ratio of 7929.41 was calculated. Patient's urinalysis one year ago showed trace protein and total protein of 34 mg/dL and creatinine of 144 mg/dL.

An abdominal ultrasound was done that was suggestive of acalculous cholecystitis. There was newly increased renal echogenicity but no hydronephrosis.

From days three to five, patient's urine output declined and serum creatinine continued to rise from 4.75 mg/dL up to 8.74 mg/dL and patient was started on Hemodialysis. Laboratory tests for different glomerulopathy causes resulted negative (Table 1).

**Figure 1:**
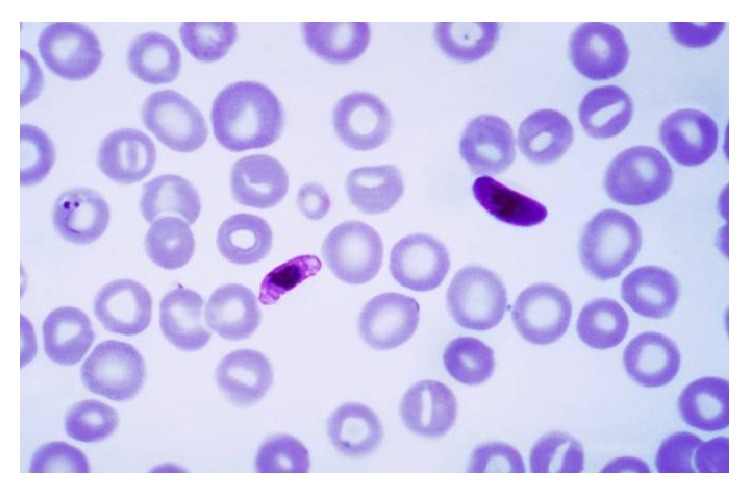
Blood peripheral smear showing crescentic shaped gametocytes.

In view of recent travel, a blood smear for malaria was done that showed crescentic shaped gametocyte which is usually the shape of* Plasmodium falciparum* and parasitemia of 1.64% (Figure 1).

Patient was then started on Coartem (Artemether: 20 mg and Lumefantrine: 120 mg) for malaria. This is considered as the treatment of acute malarial infections due to* Plasmodium falciparum* in geographical regions where chloroquine resistance has been reported.

**Figure 2:**
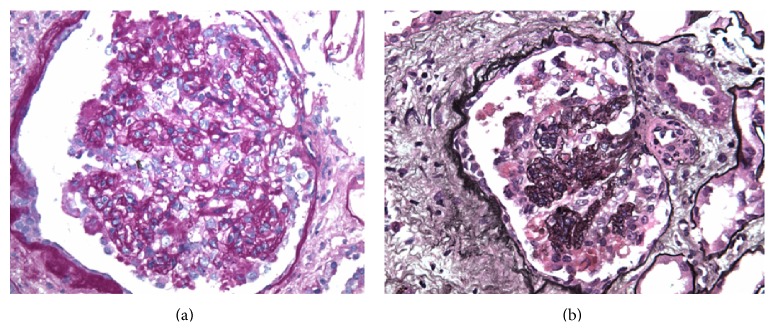
Light microscopy of the kidney biopsy. Glomeruli displayed lesions of collapsing glomerulopathy which ranged from segmental to global are shown by periodic acid-Schiff (a) and methenamine silver staining (b).

Finally, the kidney biopsy was done (Figure 2) that was consistent with severe collapsing glomerulopathy. It showed collapse of the glomerular tufts and prominent hyperplasia of overlying epithelial cells, acute injury of the tubules, interstitial fibrosis and arteriosclerosis, complete foot process effacement, and tubular degenerative changes.

Patient's symptoms improved after starting the antimalarials and he was discharged after few days, but his kidney function never returned to his baseline and he continues to use renal replacement therapy.

## 3. Discussion

We presented an interesting case of acute renal failure secondary to falciparum malaria showing histopathology of collapsing FSGS. We briefly discuss the pathogenesis, treatment, and prognosis of this case in light of the current literature review.

The pathogenesis of collapsing FSGS in patients infected with* Plasmodium falciparum* infection is not clear. It is postulated that it is similar to that of HIV associated FSGS in which the glomerular visceral (podocytes) and parietal epithelial cells sustain injury that eventually results in cellular dedifferentiation and proliferation of the epithelial cells and a loss of the glomerular filtration [1, 6, 7]. Comparing HIV to non-HIV patients, collapsing FSGS presents similarly in terms of age, sex, serum creatinine, proteinuria, and glomerular findings [8].

So far, no defined treatment exists for malaria associated FSGS. According to a study by Albaqumi et al. performed in 2006, FSGS did not respond to corticosteroid treatment in 72 % of the cases [1]. Glucocorticoid and immunosuppressive agent therapies are recommended for patients based on anecdotal evidence only, and the only proven intervention for suppressing the progression of disease process is the treatment of the underlying cause. Malaria-induced kidney injury is usually managed by antimalarials, supportive therapy, and Hemodialysis (HD), but as said before no specific treatment exists for FSGS caused by malarial infection [9].

The prognosis of FSGS not associated with HIV infection was reported to be comparatively poor especially in African patients and a progression to end-stage renal disease (ESRD) is seen despite treatment [10, 11].

Ehrich and Eke performed a meta-analysis using African data and literature on Afro-Americans about the malaria infected children undergoing renal failure in Africa. They concluded that FSGS and minimal change disease were found to be a more frequent and unusual cause of nephrotic syndrome in West Africans as compared to the rest of African regions and Europe, where membranoproliferative glomerulonephritis (MPGN) and quartan malarial nephropathy are the usual culprits [12]. This is supported by similar studies amongst African children in Ghana [13, 14]. Our patient was also born in Ghana and hence the finding of collapsing FSGS is consistent with the previous rare observations for this geographical area. This case should encourage future studies to unfold the pathophysiology of this specific phenomenon and to evaluate any specific risk factors pertaining to the parasitic features in the specific geographic area.

## Figures and Tables

**Table 1:** Glomerulopathy laboratory tests.

Antineutrophil Antibody (ANA)	Negative

Antidouble Stranded DNA (Anti-dsDNA)	Negative

Anti-GBM (Antibody to Glomerular Basement Membrane)	<1.0

ANCA (Antinuclear Cytoplasmic Antibody)	Negative

Hepatitis B and hepatitis C	Negative

HIV (Human Immunodeficiency Virus)	Negative

Complements C3, C4	Normal

Kappa/Lambda	1.61

SPEP (Serum Protein Electrophoresis)/UPEP (Urine Protein Electrophoresis)	Negative
